# Osteotomized folded scapular tip free flap for complex midfacial reconstruction

**DOI:** 10.20517/2347-9264.2021.44

**Published:** 2021-07-04

**Authors:** Catherine T. Haring, Emily J. Marchiano, Jayne R. Stevens, Kelly M. Malloy, Keith A. Casper, Mark E. Prince, Steven B. Chinn, Chaz L. Stucken, Andrew J. Rosko, Matthew E. Spector

**Affiliations:** 1 Department of Otolaryngology-Head and Neck Surgery, University of Michigan, Ann Arbor, MI 48109, USA.; 2 Tripler Army Medical Center, Honolulu, HI 96859, USA.

**Keywords:** Head and neck reconstruction, midface reconstruction, maxillary buttress, midfacial axes, osteotomized flap, scapular tip free flap

## Abstract

**Aim:**

To describe a novel technique for the reconstruction of geometrically complex defects of the midface using an osteotomized folded scapular tip-free flap.

**Methods:**

Five patients underwent maxillectomy with defects disrupting two or more of the following facial axes: orbital, nasofacial, and palatal axes. Patients underwent primary reconstruction using an angular artery-based scapular tip-free flap with an osteotomy to fold the flap. Harvest techniques, including placement of osteotomies, folding and plating, surgical esthetic, and functional outcomes, are presented.

**Results:**

Osteotomies placed in the scapular tip-free flap allowed folding of the osseous flap and improved restoration of all three facial axes with a single flap. In one patient, the tip of the scapula was used to reconstruct the nasofacial axis, while the body and lateral border were used to reconstruct the palate. In four patients, the tip of the scapula was used to reconstruct the orbital axis, while the body and lateral border were used to reconstruct the nasofacial axis. Patients had successful oronasal separation, healed wounds withstanding adjuvant therapy, satisfactory orbital positioning and facial projection, preserved masticatory surfaces and opportunity for dental implants.

**Conclusion:**

The midface is geometrically complex and is one of the most challenging head and neck sites to reconstruct. Ablative defects in this area can disrupt facial axes resulting in poor esthetic and functional outcomes. This study demonstrates the reconstructive advantages of a novel osteotomized folded scapular tip-free flap.

## INTRODUCTION

Loss of midfacial structures due to tumor extirpation, trauma, or congenital defects can have significant functional and aesthetic consequences^[1]^. Due to the complex anatomy and variable effects on form and function, midfacial reconstruction is the most challenging head and neck reconstruction. Common goals in midfacial reconstruction include restoring oronasal separation, supporting the orbit, obtaining a healed wound in preparation for possible adjuvant therapy, reestablishing facial contour, and allowing for functional dentition^[2]^. The reconstructive ladder includes the use of obturator prostheses, local, pedicled and free flaps. Bone reconstruction establishes long-term facial contour, supporting the facial envelope and globe, and has become standard for complex maxillary reconstruction^[3]^.

The radial forearm, fibula, iliac crest, and scapula osteocutaneous free flap have been described for use in boney reconstruction of maxillary defects^[2,4]^. Previous studies have demonstrated the utility of the scapula tip for midfacial reconstruction^[5–8]^. The scapula tip-free flap has advantages over the fibula or iliac crest related to the chimeric nature of the subscapular vascular system allowing for multiple tissue types, long vascular pedicle, ability to perform extirpation and flap harvest concurrently, acceptable donor site morbidity, and excellent versatility in rebuilding 3-dimentional bone structures of the craniofacial skeleton^[5,6,8,9]^. The scapula tip can be placed horizontally to reconstruct the palatoalveolar complex or vertically to restore the zygomaticomaxillary and nasomaxillary buttresses^[8,10]^. However, some authors report difficulty orienting the scapula tip bone to simultaneously reconstruct the vertical and horizontal buttresses in complex maxillary defects.

Osteotomizing the scapular tip-free flap has been described in mandibular reconstruction^[6]^. However few have described this technique and surgical outcomes in midfacial reconstruction. Here, we present five cases of complex midfacial reconstruction performed with scapular tip-free flap with an osteotomy to allow for folding of the flap and simultaneous restoration of multiple facial axes.

## METHODS

**Figure 1.**
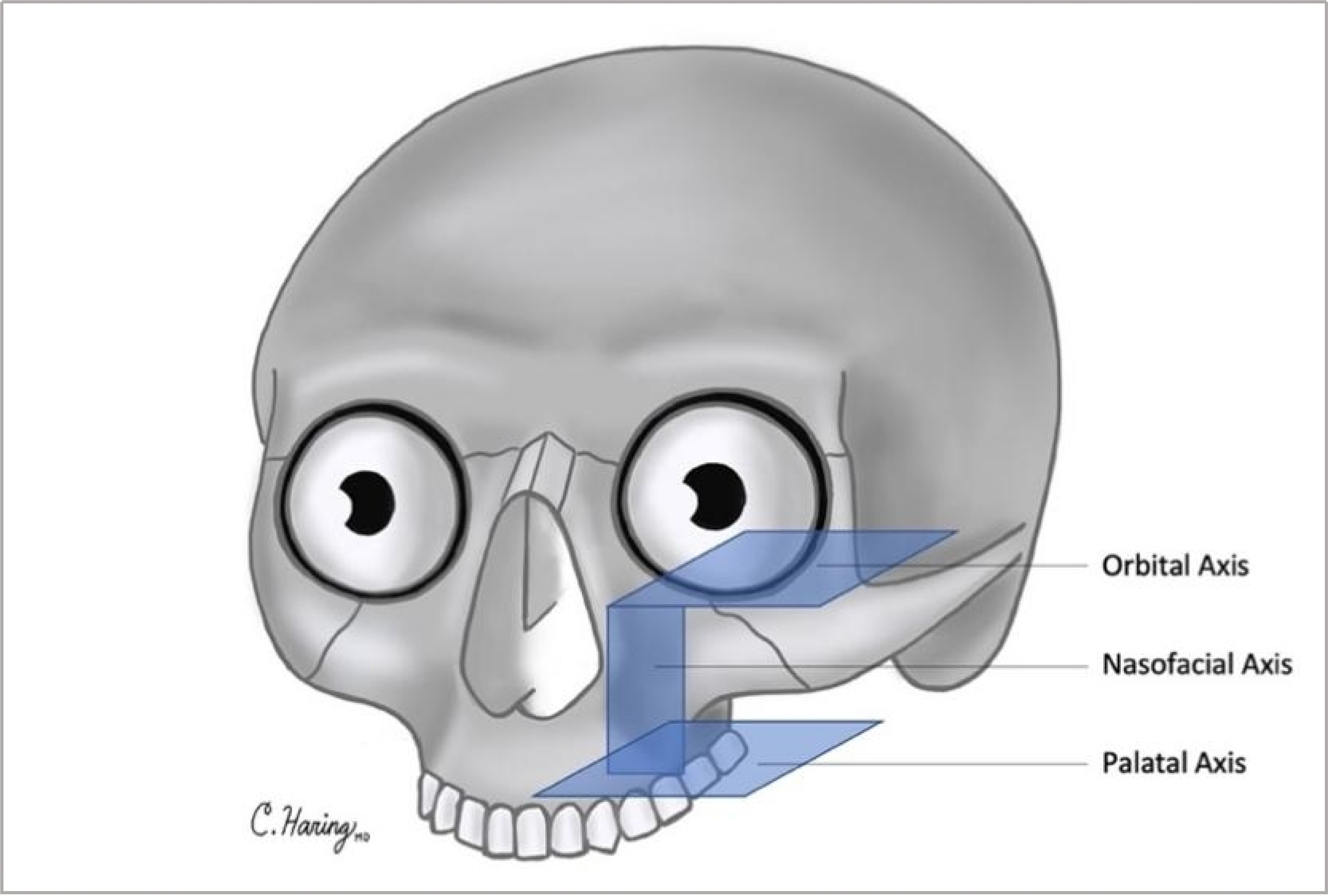
Three axes of the maxilla: orbital axis, nasofacial axis, palatal axis.

**Figure 2.**
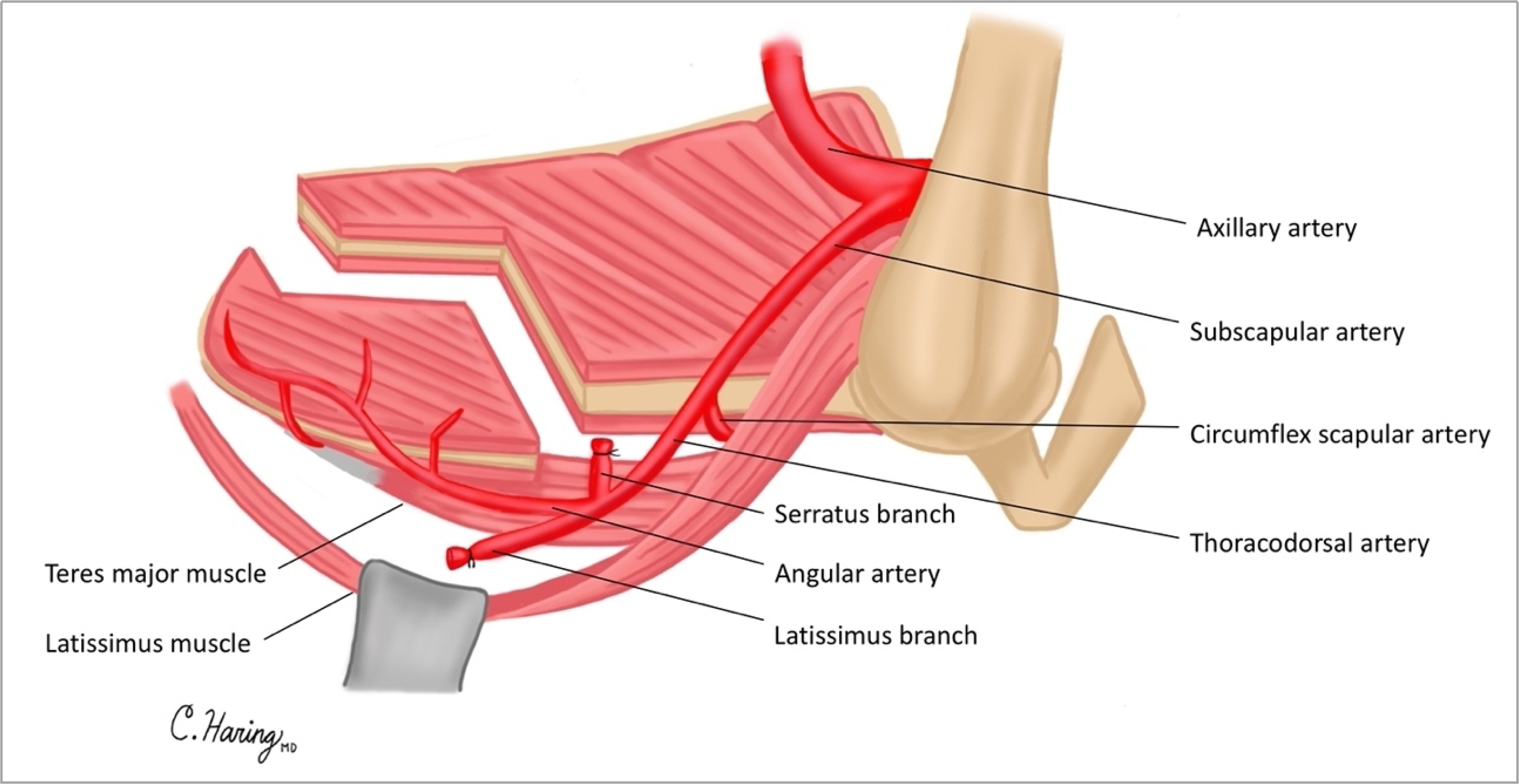
Subscapular arterial system when accessed via semidecubitous position.

This study was approved by the University of Michigan IRB (HUM 00116093). Patients underwent surgical resection of tumors, which included maxillectomy without orbital exenteration. Maxillectomy defects differed but resulted in the disruption of two or more of the following facial axes: orbital, nasofacial, and palatal axes [Figure 1]. In all cases, an angular artery-based scapular tip-free flap was harvested as previously described in a semidecubitous position using the Spider arm for optimal positioning^[11]^. Free flap harvest was performed concurrently with tumor ablation. The anatomy of the subscapular arterial system, when viewed from a semidecubitous position, is shown in Figure 2. An osteotomy was created in the scapula tip to fold and reconstruct these complex midfacial defects.

Open osteotomies were performed with a “green stick” fracture method to maintain continuity of the periosteum to ensure adequate blood supply to the distal segments. After measurement of the boney defect in both planes, the scapula is harvested in the standard fashion. The subscapularis muscle and infraspinatus muscle are cut sharply off the scapula to allow for a more precise osteotomy. There is no extra length of bone needed as the greenstick fracture of the scapula does not put the muscular attachments on tension, and there is still excellent blood flow from the angular artery to both segments. Depending on patient preference, either the left or right scapula can be used with minimal change in the pedicle and/or boney geometry. For defects where the scapular tip recreates the orbital axis, the pedicle comes off the front of the face of the maxilla and over the mandible. For defects where the scapular tip recreates the nasofacial axis or palatal axis, the pedicle comes off posteriorly and is brought through the parapharyngeal space.

## RESULTS

The demographic information, defect description, and classification of patients who underwent osteotomized folded scapula tip-free flap are included in Table 1. Folding of the scapula tip-free flap with an osteotomy allowed for simultaneous reconstruction of two or more facial axes. Mean follow-up was 2.8 years. Aesthetic and functional outcomes are listed below.

### Case 1

**Figure 3.**
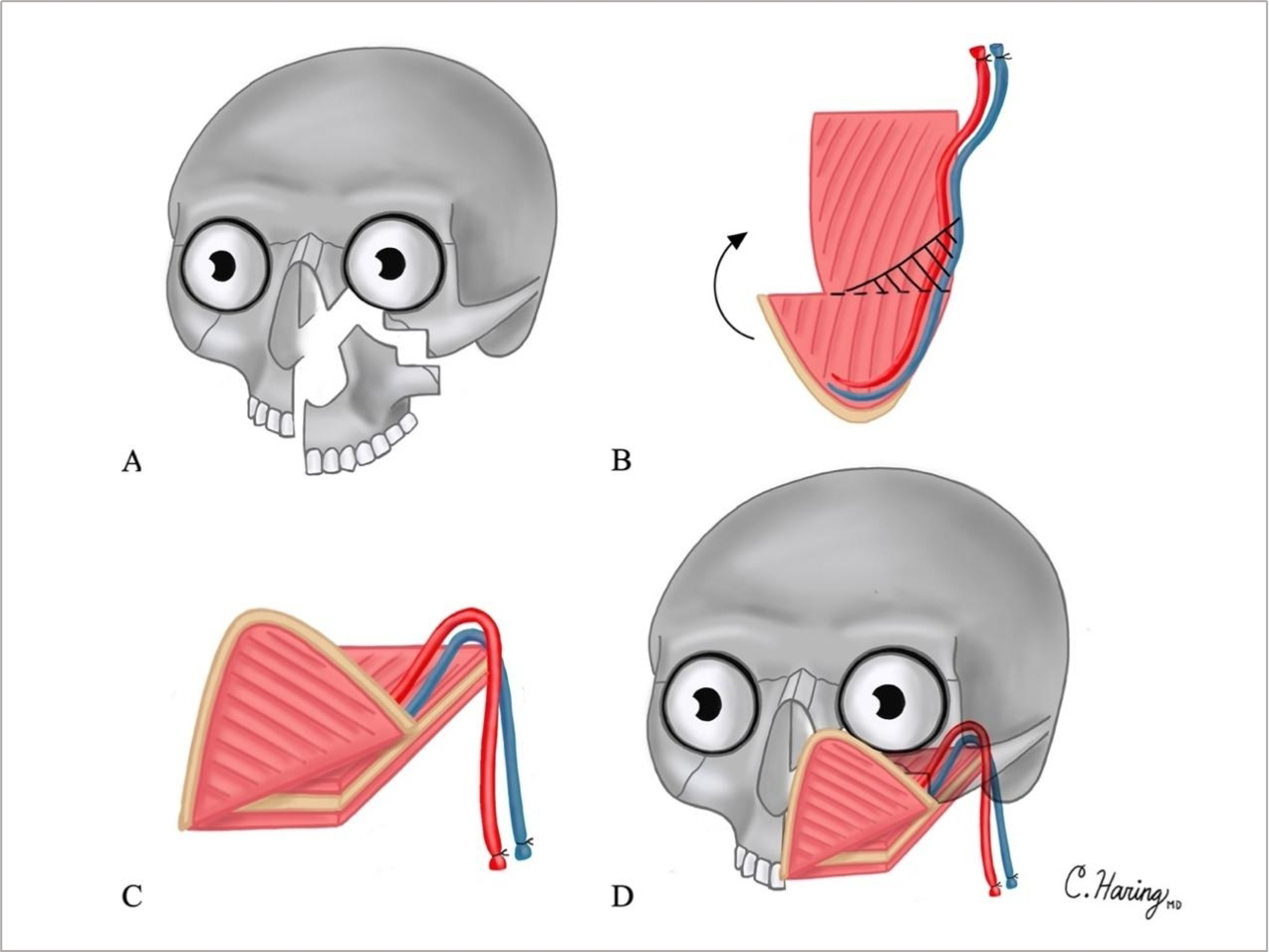
Illustrations of Case 1 surgical defect and reconstruction are shown in Figure 3A–D: (A) surgical defect involving the hemipalate, premaxilla, alveolar and frontal processes of the maxilla, and a portion of the orbital rim; (B) osteotomies created in scapula tip; shaded area represents wedge of resected bone to optimize facial contour; (C) folding of scapula tip; and (D) reconstruction of surgical defect.

A 50-year-old woman diagnosed with osteosarcoma of the left maxillary sinus was treated with neoadjuvant chemotherapy followed by maxillectomy without orbital exenteration. The surgical defect included the left hemipalate, premaxilla, alveolar, zygomatic and frontal processes of the maxilla, and a portion of the orbital rim, disrupting the nasofacial and palatal axes [Figure 3A]. Reconstruction plates were pre-bent on the patient’s 3D printed model. Plates were placed along the orbital rim and the premaxilla. The scapula tip-free flap was harvested as previously described^[11]^, and an osteotomy was created [Figure 3B]. The scapula tip was folded to reconstruct two facial axes [Figure 3C]. The tip of the scapula was used to reconstruct the premaxilla and piriform aperture, while the body and lateral border of the scapula was used to reconstruct the palate [Figure 3D]. Follow-up was over 4.5 years. The patient had successful oronasal separation, satisfactory orbital positioning, and facial projection. She successfully underwent dental rehabilitation and implantation 2 years after surgery. She underwent secondary bone grafting and anterior vestibuloplasty and received 4 endosseous implants into the scapula bone. The patient did not have cancer recurrence during the follow-up time.

### Case 2

**Figure 4.**
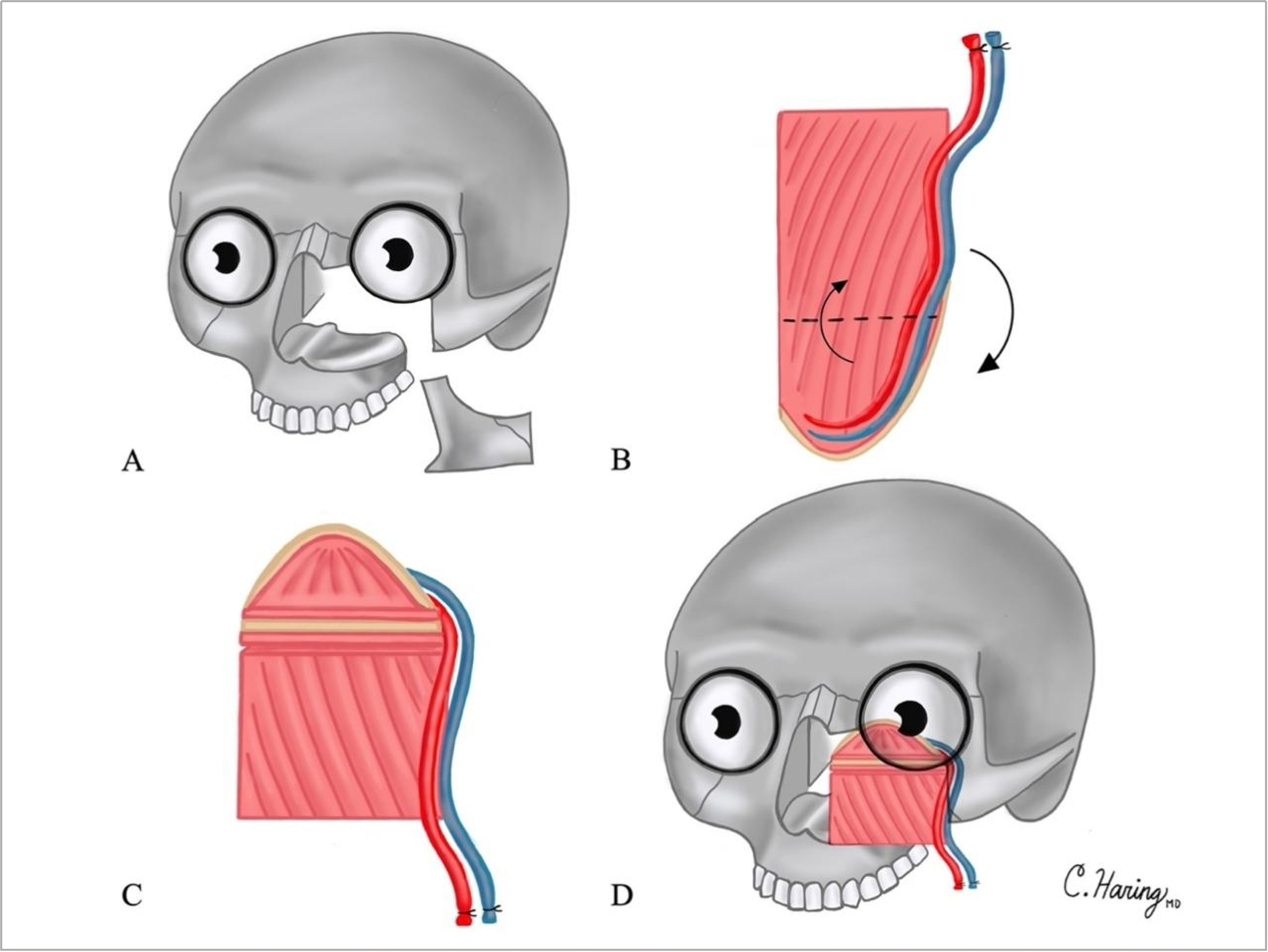
Illustrations of Case 2 surgical defect and reconstruction are shown in Figure 4A–D: (A) surgical defect including the orbital floor and rim, and superior half of the maxilla, including the frontal and zygomatic processes; (B) osteotomies created in scapula tip; (C) folding of scapula tip; and (D) reconstruction of surgical defect.

A 64-year-old man diagnosed with squamous cell carcinoma of the left maxillary sinus was treated with maxillectomy without orbital exenteration with a surgical defect including the orbital floor and rim and superior half of the maxilla, including the frontal and zygomatic processes. This resulted in disruption of the orbital and nasofacial axes [Figure 4A]. The palate was left intact. Reconstruction plates were placed along the orbital rim across the maxilla to the lateral buttress. The scapula was harvested, and an osteotomy was created [Figure 4B] to allow for folding [Figure 4C]. The tip of the scapula was used to reconstruct the orbital floor, while the body and lateral border were used to reconstruct the nasofacial axis [Figure 4D]. Follow-up was over 3.5 years. The patient had satisfactory orbital positioning without diplopia and had excellent facial projection. The palate was left intact, and the patient was able to return to normal mastication postoperatively.

### Case 3

A 29-year-old woman with osteosarcoma of the left maxilla was treated with neoadjuvant chemotherapy followed by maxillectomy without orbital exenteration and extensive buccal space resection. The surgical defect was a total maxillectomy including the left orbital floor and rim, premaxilla, alveolar and frontal processes of the maxilla, and the left hemipalate, disrupting the orbital, nasofacial and palatal axes. A reconstruction plate was placed along the orbital rim across the maxilla. The scapular tip was harvested as above, and an osteotomy was created to allow for folding. The tip of the scapula was used to reconstruct the orbital floor, while the body and lateral border were used to reconstruct the nasofacial axis, similar to Figure 4. The palate was not reconstructed, but instead, an obturator was placed at the time of surgery. A latissimus myocutaneous free flap was used to reconstruct the buccal mucosa. Follow-up was over 3 years. The patient had excellent facial projection and orbital positioning without diplopia. She had early dental rehabilitation with the use of the obturator. She did develop local recurrence one year after surgery and underwent endoscopic re-resection and adjuvant radiation.

### Case 4

Sixty-year-old man with squamous cell carcinoma of the right maxillary sinus treated with right maxillectomy without orbital exenteration. The surgical defect included the orbital floor and rim and superomedial aspect of the maxilla, including the frontal process and piriform aperture. The lateral buttress and palate remained intact. A reconstruction plate was placed along the inferior orbital rim from the nasal bones to the lateral orbital rim. The scapular tip was harvested as above, and an osteotomy was created to allow for folding. The tip of the scapula was used to reconstruct the orbital floor, while the body and lateral border were used to reconstruct the nasofacial axis, similar to Figure 4. He underwent adjuvant chemoradiation. Follow-up was over 2.5 years. The patient had excellent orbital positioning without diplopia and facial projection. The palate was left intact, and the patient was able to return to normal mastication postoperatively. He developed distant disease 1.5 years after treatment and has subsequently been treated with immunotherapy with a good response.

### Case 5

A 58-year-old man with deeply invasive basal cell carcinoma of the right facial skin and maxilla underwent maxillectomy without orbital exenteration, including facial skin. The surgical defect included the right orbital rim and floor, total maxillectomy including the alveolar and frontal processes of the maxilla, and the maxillary alveolus, disrupting the orbital, nasofacial, and palatal axes. A reconstruction plate was placed along the orbital rim. The scapular tip was harvested as above, and an osteotomy was created to allow for folding. The tip of the scapula was used to reconstruct the orbital floor, while the body and lateral border were used to reconstruct the nasofacial axis, similar to Figure 4. The cut end of the scapular body reconstructed the alveolus. A radial forearm free flap was used to reconstruct the facial skin. Follow-up was over 6 months. The patient had excellent facial projection and orbital positioning without diplopia. He returned to normal mastication postoperatively.

## DISCUSSION

Reconstruction of midfacial defects is one of the most challenging in the head and neck due to complex anatomy and variable effects on form and function. Ablative defects in this area can reduce orbital and nasal support, loss of facial projection and significant tooth-bearing segments, and formation of oronasal or oroantral fistulae. An osteotomized and folded scapula tip-free flap can be successfully used for complex midfacial reconstruction.

Alternative vascularized bone flaps include iliac crest, osteocutaneous radial forearm, and fibula free flap. The opinions on the superiority of one flap over another vary and are dependent on surgeon and institutional preference. Published literature indicates that the iliac crest free flap has been largely abandoned given donor site morbidity, including hernia development^[15]^. The osteocutaneous radial forearm has limited bone stock for significant defects but can be successfully used in smaller maxillary defects^[4]^. The fibula-free flap was historically considered the primary reconstructive option for complex midface reconstruction, given that it provides the longest bone stock and reliable bone for dental rehabilitation^[16]^. To reconstruct multiple planes of the maxilla, the fibula-free flap can be layered^[17]^; however, this results in complicated pedicle geometry and may predispose to flap complications and failure^[18,19]^.

Free flaps based on the subscapular arterial system provide the most versatile of the osseous flaps given the option for separate segments of bone, multiple skin paddles, and significant volume to fill the defect. The scapular tip-free flap from the angular artery provides a long pedicle length and has morphologic similarity to the palate^[8]^. Case series at multiple institutions have indicated that osteotomies and dental implants can be used successfully in scapular tip-free flaps for mandibular reconstruction, although there is limited data on these techniques in maxillary reconstruction^[6,20,21]^.

Here, we illustrate surgical techniques for osteotomizing an angular artery-based scapular tip-free flap for complex midfacial reconstruction. In very different maxillary defects, we describe how this technique can be used to simultaneously restore multiple planes of the 3-dimensional midface. All patients had excellent aesthetic and functional outcomes.

In conclusion, an angular artery-based scapula tip-free flap with an osteotomy can be used to reconstruct complex midfacial defects with excellent aesthetic and functional outcomes. This is a unique surgical technique that allows for reconstruction of the horizontal and vertical buttresses of the midface with a single flap. This reconstructive option allows for oronasal separation, restoration of orbital support and facial contour, and functional dentition and mastication.

## Figures and Tables

**Table 1.** Patient demographics

Case	Age/Sex	Diagnosis	Surgical defect	Follow-up	Axes disrupted	Okay *et al.*^[12]^ classification	Cordeiro and Santamaria^[13]^ classification	Brown^[14]^ classification

1	50 years/W	Osteosarcoma of left maxilla	Left orbital rim, alveolar, zygomatic and frontal processes of the maxilla, premaxilla, hemipalate	4.5 years	Nasofacial, palatal	IIf	IIIa	IIIb
2	64 years/M	Squamous cell carcinoma of the left maxillary sinus	Left orbital floor and rim, superior half of the maxilla, including the frontal and zygomatic processes	3.5 years	Orbital, nasofacial	N/a	II	V
3	19 years/W	Osteosarcoma of the left maxilla	Left orbital floor and rim, alveolar, zygomatic and frontal processes of the maxilla, premaxilla, hemipalate	3 years	Orbital, nasofacial, palatal	IIf	IIIa	IIIb
4	60 years/M	Squamous cell carcinoma of the right maxillary sinus	Right orbital floor and rim, superomedial aspect of the maxilla, including the frontal process	2.5 years	Orbital, nasofacial	N/a	II	V
5	58 years/M	Deeply invasive basal cell carcinoma of the right cheek and maxilla	Right orbital floor and rim, alveolar, zygomatic and frontal processes of maxilla, right lateral alveolus	6 months	Orbital, nasofacial, palatal	1b	IIIa	IIIb
